# Laser Shock Peening: Fundamentals and Mechanisms of Metallic Material Wear Resistance Improvement

**DOI:** 10.3390/ma17040909

**Published:** 2024-02-16

**Authors:** Xiaodie Cao, Jiali Wu, Guisheng Zhong, Jiajun Wu, Xinhui Chen

**Affiliations:** College of Engineering, Shantou University, Shantou 515063, China; 23xdcao@stu.edu.cn (X.C.); 21jlwu1@stu.edu.cn (J.W.);

**Keywords:** laser shock peening, wear resistance, conventional metallic materials, laser additively manufactured parts, laser cladding coatings

## Abstract

With the rapid development of the advanced manufacturing industry, equipment requirements are becoming increasingly stringent. Since metallic materials often present failure problems resulting from wear due to extreme service conditions, researchers have developed various methods to improve their properties. Laser shock peening (LSP) is a highly efficacious mechanical surface modification technique utilized to enhance the microstructure of the near-surface layer of metallic materials, which improves mechanical properties such as wear resistance and solves failure problems. In this work, we summarize the fundamental principles of LSP and laser-induced plasma shock waves, along with the development of this technique. In addition, exemplary cases of LSP treatment used for wear resistance improvement in metallic materials of various nature, including conventional metallic materials, laser additively manufactured parts, and laser cladding coatings, are outlined in detail. We further discuss the mechanism by which the microhardness enhancement, grain refinement, and beneficial residual stress are imparted to metallic materials by using LSP treatment, resulting in a significant improvement in wear resistance. This work serves as an important reference for researchers to further explore the fundamentals and the metallic material wear resistance enhancement mechanism of LSP.

## 1. Introduction

Metallic materials play a vital role in numerous applications, ranging from the aerospace and automotive industries to the power generation and manufacturing sectors [1]. As these materials are subjected to harsh operating conditions, such as high temperatures, corrosive environments, and mechanical stress, their mechanical properties, in particular wear resistance, become paramount to ensuring optimal performance and longevity. Therefore, there is a growing need to research and utilize effective techniques to enhance the mechanical properties of metallic components [2].

A variety of technological processes are available to improve the mechanical properties of metallic material surfaces [3], e.g., cold and hot rolling [4], shot peening [5], laser shock peening (LSP), etc. LSP has gained significant attention as a surface modification technique capable of enhancing wear resistance in metallic materials [3]. LSP involves the application of intense laser pulses to a material’s surface, generating high-pressure shock waves that induce beneficial residual stress and microstructural changes [6], where the former can significantly enhance mechanical performance, leading to improved wear resistance and a longer fatigue life [7]. Peyre et al. [8] provided a detailed overview of the current trends in physics, mechanics, and applications related to LSP. This technique enhances mechanical behavior by imparting beneficial deep compressive residual stress to metallic alloys, thereby increasing the service life of the treated specimens and preventing crack growth, wear, and stress corrosion cracking. Clauer et al. [9], in a research study on LSP, found that the use of this technique imparted a residual stress of 0.5 to 1 mm or more to the layer below the metal surface and increased the fatigue life of metallic parts. Therefore, LSP has been recognized as an effective technique for addressing wear failure in advanced manufacturing industries.

On the other hand, surface wear can occur due to microcracks or localized plastic deformation in the material resulting from the movement of surfaces in relation to one another [10]. The relationship between friction and the wear of metal surfaces is well established, with the surface properties of hardness and surface roughness, mechanical properties, and hardening behavior playing crucial roles [11]. For instance, in the research study conducted by Mikhin et al. [12], it was revealed that the friction coefficient decreased with surface microhardness. Similarly, Liu et al. [13] demonstrated that the friction coefficient varied significantly based on factors such as the shape, size, and surface hardness of the worn particles. This suggests that improving the surface properties of metallic materials could potentially reduce friction on their surfaces. LSP has been proven to be a highly effective method for enhancing surface properties. In a comprehensive review by Montross et al. [14], the authors highlighted the considerable modification of the mechanical behaviors of metals that can be achieved using LSP. The swift expansion of laser-generated plasma creates a shock wave that travels through the material, resulting in deformation and increased compressive residual stress near the surface exposed to the laser [15]. It has been confirmed that LSP has the ability to enhance surface hardness, fatigue strength, wear resistance, and anti-corrosion ability in diverse metals, such as titanium alloys, magnesium alloys, stainless steel, aluminum alloys, etc. [16,17,18,19].

In this work, we provide an overview of research on LSP, covering its fundamental principles and the mechanism related to laser-induced plasma shock waves. Additionally, we present detailed examples of wear resistance enhancement in metallic materials using LSP treatment considering different material types, i.e., conventional metals, laser additively manufactured parts, and laser cladding coatings. This work stands as an important reference for further investigations into the main mechanisms related to LSP, including wear resistance improvement in LSP-treated metallic components.

## 2. Fundamentals of LSP

The fundamental mechanisms underlying LSP involve complex physical phenomena, including shock wave generation, the material’s response to high-pressure loading, and the subsequent microstructural changes induced by the process [20]. Residual compressive stress, texture change, lattice distortion, dislocations, and grain refinement are imparted to the metal subsurface layer with LSP to enhance material hardness and wear resistance [21,22,23]. At the base of LSP are the laser action mechanism, heat conduction theory, residual stress theory, material phase change theory, and mechanical behavior theory. Briefly, the laser-induced strengthening process improves material performance through the generation of compressive stress and microstructural modifications, resulting in improved resistance to wear, deformation, and failure.

### 2.1. Principles of LSP

**Figure 1:**
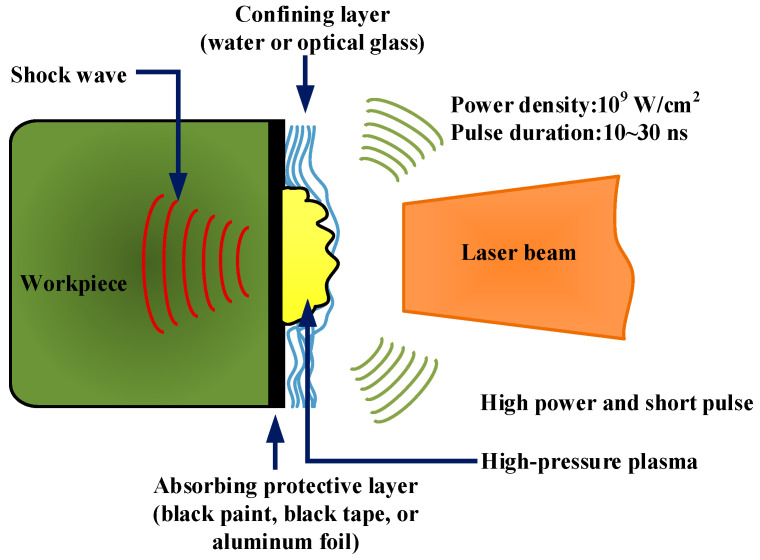
Schematic diagram of LSP process [2].

The LSP process involves directing a high-energy pulsed laser beam toward the material’s surface, leading to rapid localized heating and subsequent rapid cooling [24]. As illustrated in Figure 1, prior to being subjected to LSP, the surface of the material is coated with an absorbent protective layer (e.g., black paint, black tape, or aluminum foil) [2]. Subsequently, it is overlaid with a confining layer, such as running water or optical glass, the primary objective of which is to enhance the pulsed laser energy absorption efficiency of the metallic material or alloy while safeguarding its surface against laser thermal ablation. A high-power laser beam (10^9^ W/cm^2^) with a short pulse width (10~30 ns) can pass through the transparent confinement layer and then interact with the metallic surface [25]. The coating on the metal surface absorbs the laser energy, which causes a sharp increase in the temperature of the material almost simultaneously. As a result of explosive vaporization, the vapor particles within the absorbing protective layer concurrently generate a substantial amount of dense plasma with high temperature (>10^4^ K) and high pressure (>1 GPa) [26]. As the plasma keeps absorbing laser energy, it rapidly expands and eventually bursts, generating a high-pressure shock wave (in the GPa order), which acts on the metal surface and propagates beyond it, inside the material [27].

**Figure 2:**
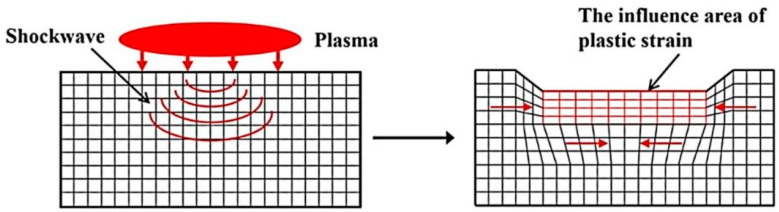
Plastic strain caused by LSP application [13].

The laser beam, characterized by a short pulse duration and high power density, penetrates the transparent boundary layer and interacts with the surface of the metallic material [28]. As a consequence of the surface being subjected to the impact of a laser-induced plasma shock wave, uniaxial stress forms in the direction of wave propagation. This, in turn, leads to plastic deformation in the LSP-affected region [29]. Once the laser-induced plasma shock wave dissipates (typically within tens of nanoseconds), the plastic deformation becomes constrained by the surrounding material. Consequently, biaxial compressive residual stress forms in a region parallel to the LSP-treated surface [30]. When materials are subjected to laser treatment, the irradiated region experiences thermal expansion. Nevertheless, upon immediate termination of laser irradiation, the material swiftly undergoes cooling and reverts back to its initial dimensions. These rapid thermal expansion and subsequent cooling processes induce significant stress and strain within the material, which potentially surpasses its elastic limit, causing plastic deformation. The process of LSP-induced plastic deformation of the material surface, as illustrated in Figure 2, can lead to the development of a desirable gradient compressive microstructure, beneficial compressive residual stress, and optimal properties within the near-surface layer.

### 2.2. Laser-Induced Plasma Shock Waves

According to the principle of LSP, the diffusion of laser-induced plasma shock waves in metallic materials can trigger a dynamic response with a high strain rate near the material surface [24], reinforcing this layer. Hence, the plasma shock waves generated by using a pulsed laser play a leading role in material hardening. In this section, we explore the theoretical model and formation process of laser-induced plasma shock waves. Plasma shock waves induced by using a laser undergo a process of formation, amplification, and decay. Their formation is a result of the chasing effect caused by compression waves, while their decay is induced by the tensile effect caused by sparse waves [30], and their amplification and attenuation arise from the intersection of compressed and rarefied waves. Laser beams with high energy density are used to induce focused ionization and electronic excitation in the target medium. These excited electrons then collide with other atoms or molecules, triggering a cascading sequence of additional ionization and excitation. As this progresses, the free electrons are accelerated due to the strength of the laser field, ultimately leading to the formation of plasma. The electrons and ions within the plasma undergo stimulation and merging processes, leading to the emission of additional energy. This phenomenon, referred to as plasma amplification, occurs due to the laser fields’ ability to initiate the accumulation and augmentation of energy [31], resulting in a localized heating effect and expansion of the plasma. These events induce fluctuations in the density and refractive index of the formed plasma, subsequently affecting the transmission of laser light. In addition, the plasma undergoes various processes, including the combination of free electrons and ions and radiation combination, resulting in energy loss and decay. The equations of the theoretical model are as follows [30]:

(1) $$\frac{\partial u}{\partial t}+u\frac{\partial u}{\partial x}+\frac{1}{\rho }\frac{\partial p}{\partial x}=0$$

(2) $$\frac{\partial p}{\partial t}+u\frac{\partial p}{\partial x}+\rho C^{2}\frac{\partial u}{\partial x}=0$$

The Hugoniot acoustic speed behind the shock wave front can be determined with the following equations:

(3) $$C^{2}=\frac{dp}{d\rho }$$

(4) $$C=v\left\{\frac{2\left(\gamma -1\right)}{{\left(\gamma +1\right)}^{2}}\left[\gamma {\left(\frac{U}{v}\right)}^{2}-{\left(\frac{v}{U}\right)}^{2}\right]+\frac{8\gamma }{{\left(\gamma +1\right)}^{2}}-1\right\}$$

where *C* represents the Hugoniot acoustic speed; *U* and *v* represent the speed of the shock wave and the acoustic speed at ambient temperature, respectively; and *γ* represents the specific heat ratio.

Without considering the phenomenon of shock wave reflection, the shock wave front can be described by the following equation [30]:

(5) $$\frac{dp}{dx}=\frac{\partial p}{\partial x}+\frac{1}{U}\frac{\partial p}{\partial t}$$

where *x* represents the position of the shock wave front; *ρ* represents the mass density; and *dp*/*dx* and *∂p*/*∂x* represent the shock amplitude variation and the pressure gradient right behind the shock wave front, respectively.

(6) $$\frac{du}{dx}=\frac{\partial u}{\partial x}+\frac{1}{U}\frac{\partial u}{\partial t}$$

The equations of the presented theoretical model are useful in research on the evolution dynamics, utility, and applicability of laser-induced plasma shock waves [30]. As detailed above, in the first phase of this phenomenon, when a high-power-density pulsed laser beam is directed toward a solid target, the affected region absorbs the laser energy, melts, and evaporates, resulting in the formation of plasma. The high heat pressure exerted by the latter causes an explosion, which, in turn, generates a shock wave in the surrounding air. The plasma shock wave rapidly increases within the pulse duration prior to decaying into a local sound wave. Furthermore, the laser energy is released from the explosion source in an energy-altering shock wave, whose prolonged duration is useful in practical applications. In summary, the laser-induced plasma shock wave phenomenon involves the utilization of a pulsed laser to subject a substance to shock, resulting in the generation of plasma; this process is facilitated by the laser beams’ desirable attributes of high energy density and short pulse width, through which they rapidly increase the temperature of the substance and induce ionization, thereby causing the transformation of its constituent atoms or molecules into a plasma state. 

**Figure 3:**
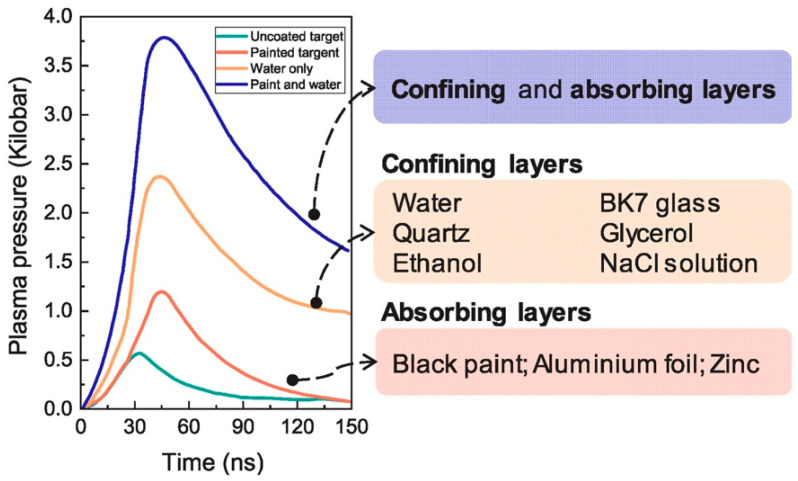
Relationship between plasma pressure and time in a laser shock wave for different LSP process configurations [7,30].

In LSP technology, the confining and absorbing layers serve as the fundamental operational variables and play a key role in guaranteeing plasma pressure exceeding the order of GPa [7]. Figure 3 presents different materials that are commonly used for these layers. Generally, glycerol and glass are applied in applications involving medium-to-high temperature or insulation demands by virtue of their insulating properties and high melting points. Currently, running water, glass, quartz, and glycerol are the main materials used for the confining layers in LSP, where glass is more suitable for processing small-scale samples in laboratory settings; running water, for large-scale LSP procedures at room temperature; and glycerol, for large-scale, high-temperature LSP processing. Table 1 provides a summary of the advantages and drawbacks of these three different confining layer materials [7].

### 2.3. Development of LSP

Since its conception, LSP has undergone a comprehensive journey, from initial research on laser-induced melting and evaporation on the material surface to widespread application across various sectors, including military, industry, materials science, and advanced manufacturing. As the demand for improved material surface properties and enhanced functionality continues to increase, LSP is emerging as a promising surface enhancement process aimed at increasing the fatigue life of metallic components [7]. Extensive research has been conducted on this topic. For instance, Montross et al. [15]. examined the importance of residual stress monitoring in the development of laser peening [32]. They described laser peening as a new surface treatment for metals, whereby cold working is used to create compressive residual stress close to the surface. Chi et al. [16] demonstrated that by using LSP, it is possible to convert residual tensile stress into compressive stress in the LAM Ti17 alloy, which greatly improves surface hardness through grain refinement and work hardening. Yang et al. [32] reported that LSP is considered a replacement technology to SP for imparting compressive residual stress to metallic alloys to improve their fatigue, wear, and corrosion resistance. Hence, LSP is emerging as a competitive alternative technology to traditional treatments to improve the fatigue life and wear resistance of metals for multiple important applications.

LSP offers noticeable technical advantages in strengthening the surface of metallic materials. In their work, Shin et al. [33] summarized some major developments in laser-based manufacturing material processing and introduced important technological issues associated with laser-based manufacturing. Among the commonly used industrial procedures covered are laser additive manufacturing, laser-assisted machining, laser micromachining, laser forming, laser surface texturing, laser welding, and laser shock peening. Processes using laser shock applications, such as LSP or laser stripping, require a deep understanding of both the mechanical and thermal loadings applied. LSP is a competitive surface-strengthening technology for post-weld treatment. In their work, Wan et al. [34] treated tungsten inert gas-welded alloy 600 joints by using LSP to enhance their mechanical properties. New experimental measurements of plasma pressure release with respect to its initial dimension were reported by Rondepierre et al. [35]; findings related to more precise plasma profiles, such as theirs, are expected to contribute to a better understanding of laser–matter interactions for laser shock applications. Zhou et al. [36] investigated the lodging of pre-coated nanopowders into the near-surface layer of IN718 SPF superalloy material by using LSP-induced GPa pressure to enhance surface hardness. Wang et al. [37] investigated the microhardness of LC-treated 30CrMnSiNi2A high-strength steel after LSP treatment, which resulted in being 25% higher than that of the substrate. In their work, Tong et al. [38] utilized the LSP technique to modify the residual stress state and microstructure of Cr-Mn-Fe-Co-Ni HEA surface layers fabricated by using laser-directed energy deposition; they found a variation in the surface residual stress state from tensile residual stress to compressive residual stress in the LSP-treated specimens, and they observed the closing of pores in the surface layers due to severe plastic deformation (SPD). In addition, it was reported that LSP led to the formation of gradient microstructures in the depth direction, which increased the strength and ductility of the LSP-treated specimens [39]. According to research by Lim et al. [40], the wear volume of 2205 duplex stainless steel material was reduced by up to 39% when LSP was used. Therefore, LSP is considered a feasible solution to reduce abrasive degradation.

In summary, LSP is a surface treatment method used to strengthen and improve the dependability of metallic parts [41]. At present, LSP technology is widely used for improving the surface properties of metallic materials as a means of improving their wear resistance, anti-corrosion properties, and fatigue life.

## 3. LSP-Induced Wear Resistance Improvement in Metallic Materials

### 3.1. Wear Resistance Improvement in Conventional Metallic Materials

Conventional metallic materials, which have wide applications in various industries, are manufactured by using traditional methods, which include traditional manufacturing processes such as casting [11], forging [19], and forming. Conventional metallic parts inevitably present surface roughness and inhomogeneity, which lead to wear and tear during use, ultimately resulting in insufficient wear resistance to meet working requirements [42].

Cast aluminum–silicon alloys are extensively applied in many fields, such as in the automotive, aerospace, and electronics sectors, due to their low mass density, good mechanical properties, thermal conductivity, machinability, etc. [11]. However, cast parts typically exhibit diminished density and an irregular organizational structure, rendering them vulnerable to wear and damage. Numerous studies have explored the wear mechanism and response of cast aluminum–silicon alloys using various surface-strengthening techniques. Park et al. [10] conducted an investigation into the impact wear behavior of LSP-treated cast aluminum–silicon alloys. In their experiment, the laser intensities ranged from 1 GW/cm^2^ to 7 GW/cm^2^, with an overlapping ratio of 50%. The laser beam spot diameter of 2.06 mm was chosen for obtaining laser irradiance within the range of 1~4 GW/cm^2^, whereas a 1.64 mm spot diameter was chosen for obtaining higher laser irradiance, within the range of 4~7 GW/cm^2^; the laser pulse duration was 10 ns. After LSP treatment, the friction of AC8A specimens (Hansin metal, Republic of Busan, Korea) was tested before and after LSP treatment. 

**Figure 4:**
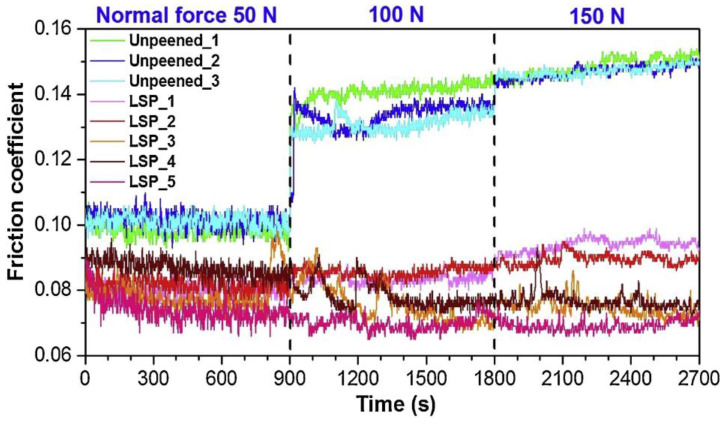
COFs of cast experimental specimens under different loading conditions [10].

The tribological properties of a material’s surface, specifically wear resistance and friction properties [43], determine its proper functioning. The coefficient of friction (COF) is a key parameter describing the friction characteristics between a material and other surfaces [44]. In Park et al.’s study, the COFs of untreated and LSP-treated samples were measured under different loading conditions, as illustrated in Figure 4. For the five laser-peened samples, the average friction coefficients (with corresponding RSDs) were measured as 0.08 (5.2%), 0.079 (7.1%), and 0.081 (11.5%) under the applied loads of 50 N, 100 N, and 150 N, respectively. These values show reductions of 20%, 42%, and 45%, respectively, compared with the friction coefficients observed in the untreated samples under the same loading conditions. In contrast to the untreated samples, which exhibited an increase in the friction coefficient with the increase in the load (e.g., from 50 N to 100 N), the friction coefficients of the laser-peened samples remained relatively stable under all tested loading conditions. This consistent response highlights the efficacy of LSP in decreasing friction.

**Figure 5:**
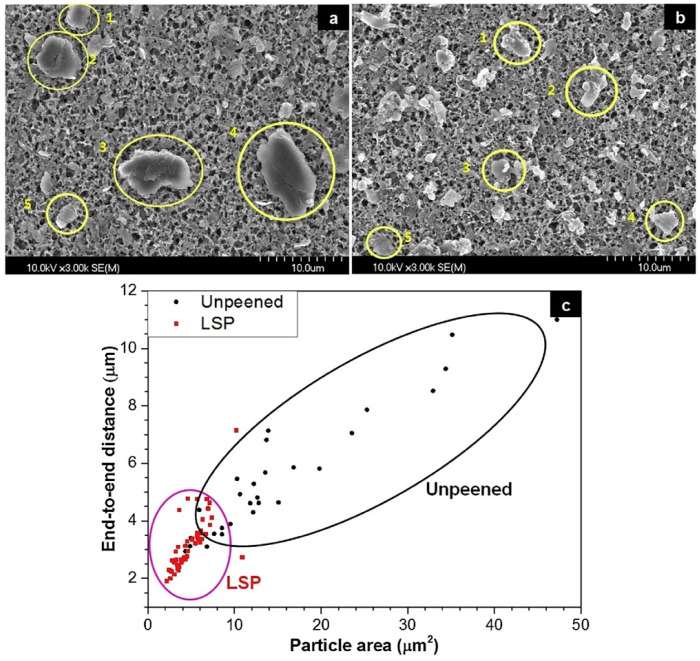
SEM images of (**a**) untreated and (**b**) LSP-treated cast experimental specimens after friction test and (**c**) their size distribution [10].

Since it is known that the size of wear particles is tightly connected to friction [11], scanning electron microscope (SEM) images of untreated and LSP-treated experimental samples were obtained, and representative samples are illustrated in Figure 5a,b, respectively. The farthest end-to-end distances and projected areas of the five largest particles on each SEM image were measured: a total of 30 particles for the unenhanced sample and 50 particles for the laser-enhanced sample. The presented images demonstrate a noticeable disparity in size between the wear particles of the laser-peened experimental samples and those of the untreated specimens. Specifically, the former exhibit significantly reduced dimensions compared with their counterparts. The average farthest end-to-end distance among the obtained wear particles in the LSP-treated experimental samples was calculated to be 3.23 μm, and the projection area, 4.90 μm^2^; in the untreated samples, the corresponding values were 5.62 μm and 16.64 μm^2^, respectively. To allow for a comprehensive understanding of the size distribution, the wear particles are graphically represented in Figure 5c, which illustrates the distinctively smaller size and size resemblance of wear particles obtained with LSP. Given the significant association between wear particle size and friction, a smaller wear particle size, which can be obtained with LSP, results in an increase in surface hardness and a reduction in surface roughness, which, in turn, have a favorable effect on the reduction in the friction coefficient and consequent improvement in wear resistance.

Table 2 summarizes the mass reductions in specimens before and after both LSP treatment and the friction test. The untreated samples exhibited a remarkable decrease in average mass loss compared with the LSP-treated samples. This outcome clearly illustrates the substantial enhancement in wear resistance in aluminum–silicon alloys achievable with LSP.

**Figure 6:**
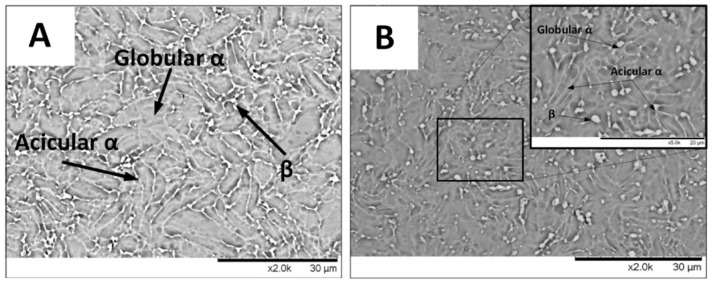
SEM images of Ti-6Al-7Nb specimen: (**A**) before LSP treatment; (**B**) after LSP treatment [45].

Besides cast aluminum–silicon alloys, forged parts have also been improved in terms of wear resistance by using LSP treatment. Forged components typically have high strength and toughness, but their surface is prone to wear and corrosion [44]. The wear resistance of titanium alloys treated with LSP was investigated by Shen et al. [45]. The laser parameters applied in their work were 6.5 J laser pulse energy, 1064 nm wavelength, 20 ns pulse width, 3 mm laser beam spot diameter, 11.5 GW/cm^2^ laser power density, and 65% overlap rate. The SEM images of the experimental specimens treated by using LSP are shown in Figure 6. Based on the SEM analysis results, prior to treatment, the microstructure of the Ti-6Al-7Nb alloy comprised globular and acicular α-phases, along with some β-phases, with non-uniform distribution, leading to the presence of coarse-grained regions with well-defined phase boundaries. On the other hand, the microscopic observation of the LSP-treated samples revealed that the grain size had decreased to 10~15 μm as a consequence of the spontaneous generation of shock waves, which induces SPD under high strain rates [46]. Consequently, globular and acicular α-phases were distributed evenly, and the grain boundaries exhibited a blurred appearance. The findings suggest that the reduced grain size and homogeneous phase distribution obtained with laser shock treatment increase the hardness of a material. In addition, LSP leads to an increase in dislocation density and induces residual compressive stress. A crystalline phase transition alters the crystalline structure of the material, while residual stress can create a layer of compressive stress. Both of these factors concurrently enhance the hardness and wear resistance of the material.

The microhardness values of Ti-6Al-7Nb before and after LSP treatment are shown in Table 3. Microhardness affects wear resistance, and high hardness can effectively improve wear resistance and fatigue life [47]. In the above study, surface hardness increased from 310 HV to 363.2 HV after one LSP pulse. With an increase in pulses by two and three times, this value increased to 367.5 HV and 372.2 HV, or by 20.6% and 22.31%, respectively. However, the improvement in work hardening depth induced by LSP treatment was limited, with the affected layer being approximately 0.9 mm deep.

**Figure 7:**
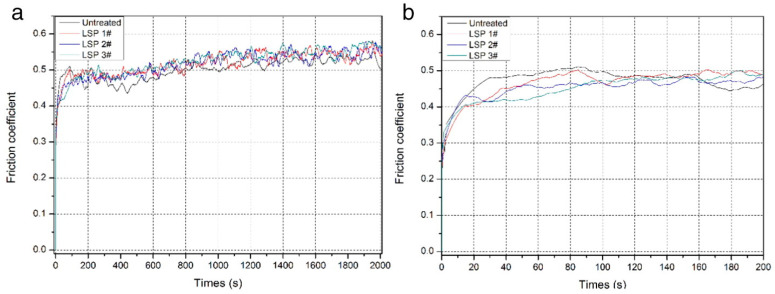
Variation in friction coefficients of Ti-6Al-7Nb specimens with time: (**a**) 0 s to 2000 s and (**b**) magnified image of the period of 0 s to 200 s [45].

According to Figure 7, it is evident that the COFs of the as-received samples increased more slowly during the first stage (0~25 s) in comparison to the treated samples and subsequently rapidly reached a high, stable value of 0.48; on the other hand, the friction coefficients of treated Ti-6Al-7Nb were reduced thanks to LSP treatment.

**Figure 8:**
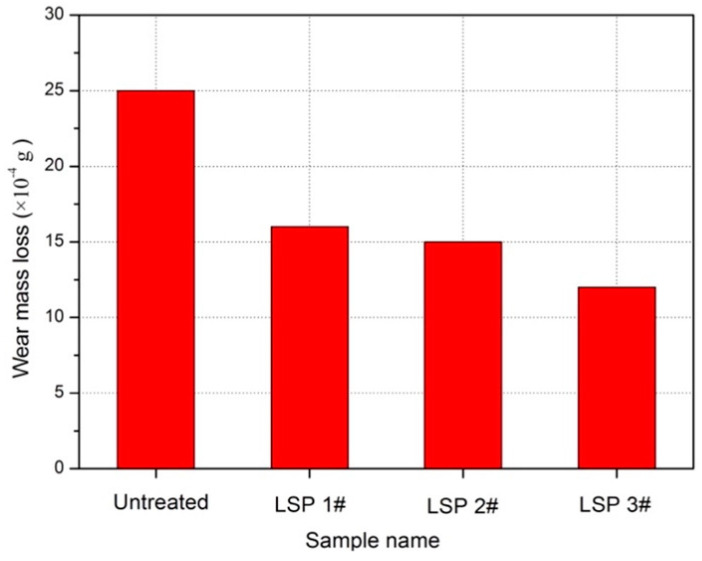
Wear mass loss results of Ti-6Al-7Nb experimental samples [45].

Investigating the effect of LSP treatment on wear resistance in a material requires examining alterations in the wear mass loss of the experimental specimen during the wear process. Figure 8 presents the comparison of wear mass reduction in Ti-6Al-7Nb alloy specimens after wear experiments. It is evident that the weight reduction in the treated specimens was lower than that in the untreated samples. This decline showed a downward trend as the number of pulses increased. Therefore, the use of LSP treatment can greatly enhance wear resistance in Ti-6Al-7Nb. The wear mass loss of the material was reduced to different degrees after LSP treatment, where the higher the number of pulses, the greater the decrease in wear mass loss.

In summary, the application of LSP enhances the grain boundary structure of cast and forged components, resulting in grain refinement and increased density of the grain boundaries [48]. Consequently, this process leads to improvements in material properties such as microhardness, strength, and wear resistance.

### 3.2. Wear Resistance Improvement in Laser Additively Manufactured Parts

The desirable mechanical properties, wear resistance, and stress corrosion resistance of laser additively manufactured parts have led to their extensive use in the domains of aircraft components, marine facilities, and petroleum and chemical sectors [49,50]. The term additive manufacturing (AM) refers to the process of building 3D parts layer by layer by using malleable materials (plastics, metals, etc.) [51].

By virtue of its usefulness in the creation of parts with complex geometries, AM has recently gained a lot of attention [52]. Additively manufactured parts typically consist of multiple layers of stacked molten metal, whose surfaces are prone to cracking and wear [53]. Consequently, wear resistance tends to be low in AM metals. Many researchers have proved that laser processing has substantial potential for improving surface characteristics in steel additively manufactured parts [54]. For instance, the mechanical properties, surface morphology, microstructural change, and wear behavior of experimental samples of selective laser melting (SLM)-treated 15-5PH stainless steel subjected to LSP treatment were investigated by Wu et al. [21]. In their study, first, 15-5PH stainless steel experimental specimens were produced by using selective laser melting (SLM). Afterward, the LSP process was employed to enhance their mechanical characteristics and wear resistance. The laser parameters applied in this work were 6 J laser energy, 3 mm laser beam spot diameter, and 15 ns pulse width.

**Figure 9:**
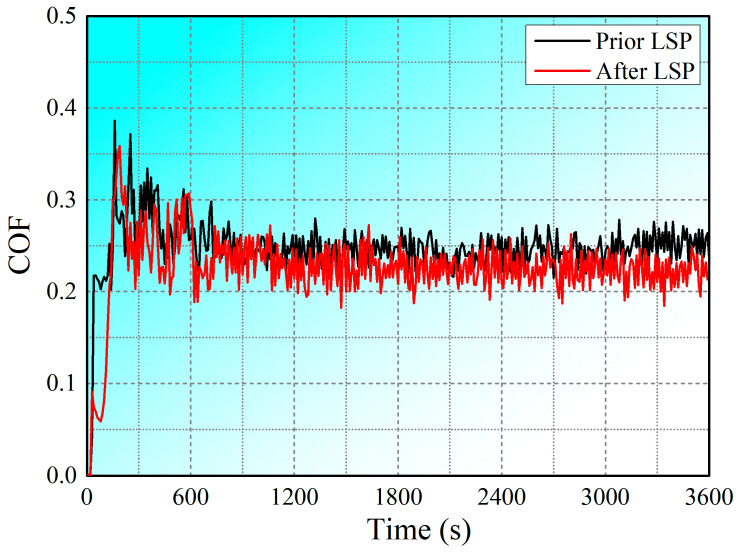
COF values of SLM-treated 15-5PH stainless steel specimens before and after LSP treatment [21].

Given that the COF and wear rate are significant factors in evaluating the wear properties of metallic materials [55], the variations in these characteristics over time were analyzed. The COF curves for SLM-treated 15-5PH stainless steel experimental samples before and after LSP treatment are shown in Figure 9. Two stages can be observed in COF curve changes: a primary break-in phase and a progressive stable phase. As the wear time increases, the curves move towards the steady phase, indicating that the COFs have stabilized, with fluctuations lying within a specific range [56]. Prior to LSP treatment, the highest COF value was found to be approximately 0.248. The average COF of the samples was reduced to 0.226 following LSP treatment, demonstrating a significant enhancement in wear resistance. Therefore, these findings indicate that LSP treatment is critical to reducing the COF by inducing plastic distortion in the near-surface layer of a material.

**Figure 10:**
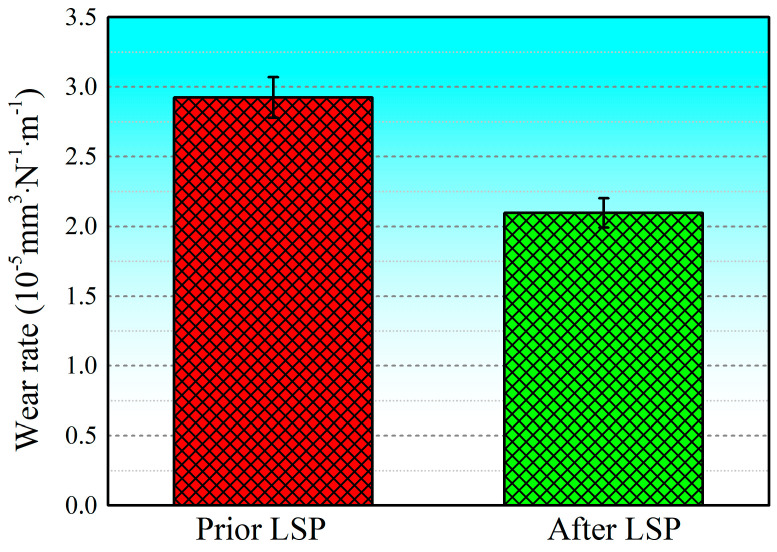
Wear rates of SLM-treated 15-5PH stainless steel specimens before and after LSP treatment [21].

Figure 10 shows the wear rates of SLM-treated 15-5PH stainless steel specimens before and after LSP treatment. The obtained outcomes demonstrate that the average wear rate of the experimental specimens before LSP treatment was 2.92366 × 10^−5^ mm^3^·N^−1^·m^−1^. However, following the application of LSP, it was reduced to 2.097311 × 10^−5^ mm^3^·N^−1^·m^−1^, a decrease of about 28.26%. This indicates that the wear resistance of SLM-treated 15-5PH stainless steel was significantly improved after LSP treatment. As the affected surface of the specimen was ground and polished prior to LSP, its initial surface state presented low surface roughness (0.275 μm), i.e., it was relatively smooth. After LSP treatment, the surface roughness of the material increased to a certain extent, to about 2.515 μm. The material’s near-surface layer experienced significant plastic deformation due to the laser-induced plasma shock wave, which caused the surface morphology to change and its surface roughness to increase [57]. Thus, the significant enhancement in wear resistance in the experimental specimens achieved by using LSP is ascribed to the refinement of the grains and the beneficial compressive residual stress layer formation, resulting in the hardening of the near-surface layer up to a specific depth.

Table 4 presents the residual stress and microhardness analysis of SLM-treated 15-5PH stainless steel before and after LSP treatment. The near-surface layer exhibited the presence of tensile residual stress, reaching a peak value of 122 MPa at the surface. This stress gradually diminished with the increase in depth, which is potentially attributed to the significant thermal gradients and rapid cooling experienced during the SLM fabrication process. Following LSP treatment, consistent and compressive residual stress was observed, reaching a maximum value of 340 MPa in the surface layer. The measurement of microhardness is essential to comprehending the mechanisms of elastic and plastic deformation in materials that have undergone LSP treatment. In the case of SLM-treated 15-5PH stainless steel, the application of LSP treatment resulted in a notable 13.07% enhancement in surface microhardness, reaching a maximum value of 422.3 HV. It is worth noting that the thickness of the hardened layer exceeded that of the compressive residual stress layer, which may be attributed to the generation of tensile residual stress during the SLM fabrication process and subsequent removal of the electrolytic layer.

**Figure 11:**
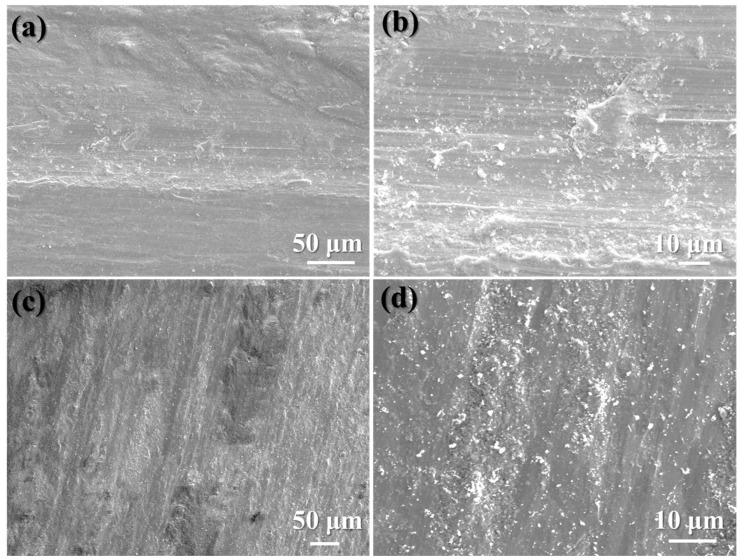
Classical wear morphologies of SLM-treated 15-5PH stainless steel experimental specimens: (**a**,**b**) before LSP treatment; (**c**,**d**) after LSP treatment [21].

Compared with the classical wear morphologies of SLM-treated 15-5PH stainless steel before LSP treatment (Figure 11a,b), the surface roughness of specimens after LSP treatment was decreased significantly (Figure 11c,d). Furthermore, the surface of the experimental specimens exhibited a rough texture with plenty of patches and wider and deeper grooves, indicating more severe damage prior to LSP treatment. Additionally, under alternating contact stress, flake-like wear debris detached from the specimens’ surface, suggesting that peeling was the primary wear mechanism in the experimental specimens prior to LSP treatment. In contrast, the sample that had undergone LSP treatment displayed plowing grooves decorated with a substantial amount of small-flake wear debris, indicative of the standard adhesive wear mechanism [58]. Hence, it can be seen that wear resistance improved following LSP treatment in the experimental samples, which aligns with the COF curve and the change in wear rate.

### 3.3. Wear Resistance Improvement in Laser Cladding Coatings

Laser cladding (LC) is a surface treatment technique that includes the deposition of a protective layer onto a substrate using a laser beam. This process offers numerous advantages, such as accurate control over the coating composition, minimal heat-affected zones, and the ability to reinforce the substrate’s surface properties. Therefore, coatings are often used to provide a protective layer and enhance surface properties, but they are susceptible to flaking and abrasion.

Several researchers have reported that using laser cladding coatings is a highly effective technique for enhancing the toughness and wear resistance of materials. For instance, Lu et al. [59] researched the LSP-induced modification in the wear properties of H13 tool steel with laser cladding Ni25 coating. In their work, in the LC procedure employed to manufacture the Ni25cladding layer, the laser parameters were as follows: the laser power was set at 2000 W; the overlap rate was 60%; the scanning velocity was 400 mm/min; and the powder-feeding velocity was 15 g/min. The LSP treatment parameters were as follows: pulsed laser duration of 10 ns, rated power of 7.6 J, and laser beam spot diameter of 3 mm. For simplicity, the Ni25 cladding coatings fabricated by using LC are designated as LC-treated experimental specimens, while the LC-treated experimental specimens further treated with LSP are denoted as LSP-treated experimental specimens.

**Figure 12:**
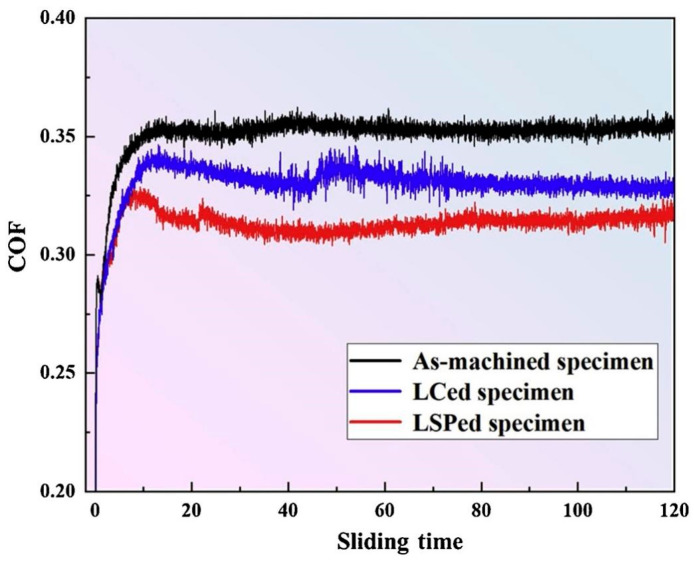
COFs of as-machined specimens, LC-treated specimens, and LSP-treated specimens [59].

The COF curves depicted in Figure 12 illustrate the behavior of the three different types of test specimens. The as-machined specimens exhibited the maximum COFs, with an average value of 0.352, while the COFs of the LC-treated samples were relatively lower, 0.331 on average. Following the application of LSP treatment, the average COF decreased to 0.313. During the early stages of the wear test, the refinement of the grain structure and the formation of a hardened layer, induced by LSP treatment, enhanced the surface wear resistance of the Ni25 coating. Nonetheless, as the wear depth increased and wear debris started to peel off, the hardness and strength of the coating gradually decreased, resulting in a reduction in wear resistance and an associated increase in the COF value. The main wear mechanism observed on the surface of the coated parts was flaking.

**Figure 13:**
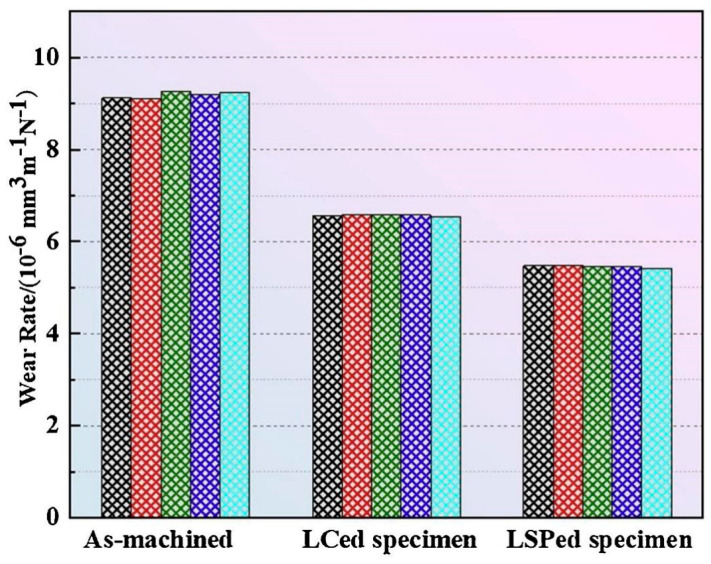
Wear rate comparison of as-machined specimens, LC-treated specimens, and LSP-treated specimens [59].

The hardness values were measured at 0.1 mm intervals along the depth five times. In order to conduct a more comprehensive analysis of wear performance, the wear rates of the three specimen types were computed, and the results are depicted in Figure 13. The underlying material still demonstrated the maximum wear rate, measuring 9.1843 × 10^6^ mm^3^ N^−1^·m^−1^. In contrast, the LC-treated samples and LSP-treated samples exhibited average wear rates of 6.5756 × 10^6^ mm^3^ N^−1^·m^−1^ and 5.4592 × 10^6^ mm^3^ N^−1^·m^−1^, respectively. The wear loss of the coating during the wear process was effectively reduced as a result of the LSP treatment, as evidenced by decreases of 29.0% and 41.1% compared with the as-machined and LC-treated specimens. There was evidence of an SPD layer following LSP application, leading to the emergence of abundant dislocation structures. As a consequence, a shift in grain type occurred, accompanied by additional grain refinement.

Table 5 shows the distributions of residual stress and microhardness in the specimens, with LC-treated experimental specimens and LSP-treated experimental specimens exhibiting depth-dependent variations. The application of high heat and the subsequent rapid cooling during the cladding process induced elevated tensile stress levels, thereby exacerbating the propensity for coating fracture. Conversely, the implementation of LSP resulted in the formation of a high-level compressive stress layer, characterized by a peak value of −453 MPa. However, it is important to note that at depths ranging from 0 to 0.5 mm, the formation of a high-density compressive residual stress layer was compromised due to the influence of laser shock wave effects, thereby weakening the overall effectiveness of this technique. LC-treated specimens showed a peak microhardness value of 432 HV at the upper surface, which gradually decreased with the increase in depth. The LSP treatment induced a significant increase in microhardness, of approximately 100 HV, with a peak value of 550 HV having been recorded at the coating surface. The increase in microhardness and residual stress was particularly pronounced at depths greater than 0.3 mm.

**Figure 14:**
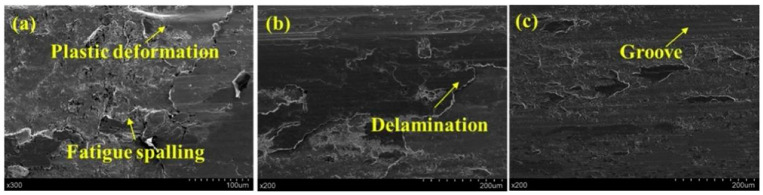
SEM images of wear morphology. (**a**) As-machined experimental specimen, (**b**) LC-treated experimental specimen, and (**c**) LSP-treated experimental specimen [59].

Figure 14 illustrates the microstructural differences among the three types of specimens. Significant plastic deformation and extensive fatigue spalling were visible in the as-machined experimental specimens (Figure 14a), showing severe adhesive wear and tear. In contrast, in LC-treated specimens, flaking pits with substantial deformations were not observed (Figure 14b). Despite some large spalling scales, the spalling depth reached by the grinding ring was clearly reduced. Furthermore, the sliding wear process resulted in the preservation of multiple small sections of the original coating. This observation suggests an increment in wear resistance in the coated surface following LSP treatment. The surface profile of the LSP-treated experimental specimens (Figure 14c) also revealed the presence of several parallel grooves, in agreement with the abrasive wear mode described by Fu et al. [60]. Accordingly, it can be deduced that the grinding ring’s compressive effect on the contact surface was successfully reduced, thus leading to a decrease in adhesive wear. Additionally, the LSP-treated experimental specimens’ wear mechanism had shifted to a combination of abrasive and adhesive wear, resulting in an overall enhancement in wear resistance.

In conclusion, the plasma shock wave generated by using a pulsed laser resulted in the refinement of the grain size on the surface of the LC-treated samples, a significant increase in low-angle grain boundaries within the surface layer, and a reduction in the length of austenite grains from 30~40 μm in the deep layer to 4~8 μm in the surface layer. The application of LSP resulted in enhanced surface hardness and abrasion resistance in the laser cladding coatings, thereby mitigating the occurrence of coating flaking and wear [61,62,63]. Additionally, according to surface characterization, the LSP treatment improved the bond between the coating and the substrate, thereby augmenting the adhesion and wear resistance of the coating.

## 4. Discussion

Grain size, microhardness, and surface roughness play key roles in determining wear resistance [64]. In this paper, we summarize the changes in wear resistance in conventional, additively manufactured, and coated components following LSP treatment, including changes in surface roughness, microhardness, and residual stress, which determine the wear properties of the COF, wear rate, and wear mechanism. The laser shock pressure generated with LSP greatly surpasses the Hugoniot elastic limit of solidified metal at elevated temperatures [65], thereby causing plastic deformation in the outer layer of the metal surface subjected to treatment [66]. SPD occurs near the surface of the material when the original sample is very smooth, indicating that LSP can greatly increase surface roughness. Generally, lower surface roughness results in lower COF and wear rate [67]. However, LSP treatment has been observed to clearly reduce the COFs and wear rates of conventional metallic materials, additively manufactured materials, and coated materials (Figure 9, Figure 10, Figure 12, andFigure 13). LSP treatment induces significant, deep, beneficial compressive residual stress in the near-surface layer, which is discharged during wear, preventing plastic deformation. Following LSP treatment, the material experiences a considerable increase in microhardness, which reduces the negative effect of increased surface roughness on the material’s wear resistance, improving the latter. The pulsed laser in LSP induces the surface effect of cold work hardening on forged components, thereby enhancing the material’s grain boundary continuity and grain refinement [68]. According to the results reported in Figure 6, following LSP treatment, the surface of cast components becomes denser and more uniform, improving the crystalline structure, eliminating internal defects, and increasing the continuity of grain boundaries. Consequently, in accordance with the changes in wear morphology in specimens before and after LSP treatment, this strengthening technique effectively improves wear resistance in metallic materials.

**Figure 15:**
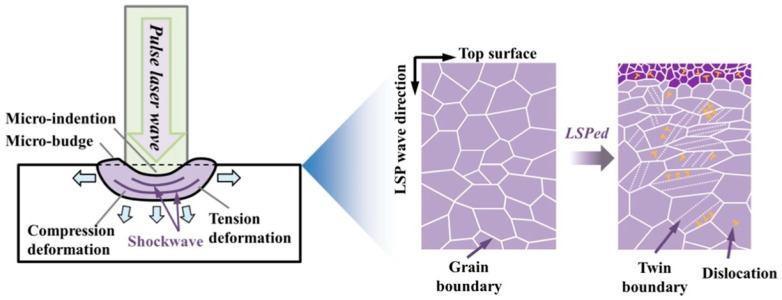
Schematic of microstructural change in metallic materials treated by using LSP [70].

The main forms of plastic deformation in metals involve dislocation slip and deformation twinning. As shown in Figure 15, pulsed laser irradiation generates a shock wave, imparting both tensile and compressive deformation to the layer near the material surface. Additionally, LSP application causes the formation of micro-indentations and micro-bulges on the material surface. Therefore, the LSP treatment method enhances the grain boundary structure, promotes grain refinement, and increases surface hardness and wear resistance [69]. Specifically, the application of LSP to the target surface, i.e., the directing of repeated high-intensity laser pulses at the same location, results in the generation of a deformation zone (DZ) presenting both compressive and tension deformations (Figure 15). Consequently, the wear mechanism of the material is altered, resulting in a reduction in the incidence of abrasive wear and fatigue wear.

In LSP, the high-density energy affects grain orientation and triggers grain slip, resulting in a significant number of dislocations [70]. Dislocations multiply and result in grains moving around in bulk materials, as well as near the surface. The concentration of these dislocations within the material restricts grain slip, causing grain refinement. This, in turn, contributes to the enhancement in the mechanical properties of the material.

According to the Hall–Petch theory, the relationship between the microhardness and grain size of a material can be indicated as [60]

(7) $$H=H_{m}+\alpha Gb\rho$$

where *H* is the microhardness, *H_m_* is the initial microhardness, *α* is the material’s constant, *G* is the shear modulus, *b* is the Burgers vector, and *ρ* is the dislocation density. According to Equation (7), an enhancement in the density of dislocations results in greater microhardness. Following the LSP process, a substantial plastic deformation layer and numerous dislocation structures are generated, leading to a modification in the grain type and further grain refinement [71]. According to the results in Figure 12 and Figure 13, both the coefficient of friction and the wear rate of the coatings after LSP treatment declined significantly, and evidence of the abrasive wear mechanism was found. Following the application of LSP treatment, small-sized carbides are effectively joined with the matrix by the shock wave, thereby acting as a barrier against deformation and enhancing wear resistance [72]. In the region affected by the shock wave, the grain size reduces, leading to a substantial increase in the quantity of grain boundaries [73]. Additionally, LSP-treated cladding parts are more prone to adhesive wear than to peeling wear, unlike untreated materials [74].

Under high-strain-rate conditions, the response of materials can be significantly different from that under low-strain-rate conditions. Adiabatic shear bands (ASBs) are narrow localized bands of intense plastic deformation that form due to severe shear stress imposed on the material [75]. In the context of dislocation slip and twinning, ASBs play a crucial role in the deformation of HCP materials. Grain refinement is regarded as the optimal mode of plastic deformation during the LSP process, and the density of dislocations is calculated as follows [21]:

(8) $$\rho =\frac{\Delta \theta }{db}$$

where *ρ* is the shear strain within the adiabatic shear band; Δ*θ* is the change in angle between adjacent crystallographic planes; *d* is the spacing between the crystallographic planes across which the shear deformation occurs; and *b* is the magnitude of the Burgers vector, which characterizes the magnitude and direction of the dislocation slip or twinning. Microhardness and wear resistance are well known to be correlated, and their relation is described as follows [59]:.

(9) $$V=K\frac{PL}{H_{V}}$$

where *V* represents the amount of wear, *K* represents the wear coefficient, *P* represents the load, *L* represents the sliding distance, and *H_v_* represents the material’s microhardness. Based on Equation (9), an increase in microhardness reduces weight loss. According to the results in Figure 10 and Figure 13, LSP-treated additively manufactured and coated parts present a reduced wear rate and increased wear resistance. The collective reinforcement effect induced with LSP is ascribed to the strengthening mechanisms of fine-grain formation and dislocation strengthening [76]. These mechanisms not only improve the microhardness of the coating but also increase wear resistance.

During surface hardening, plastic strain and residual stress induce a modified von Mises stress state. The LSP-induced modification of the surface layer effectively enhances the microhardness of the material. Hence, the enhancement in wear resistance in conventional metallic materials, laser additively manufactured parts, and laser cladding coatings through LSP treatment is due to the improvement in wear patterns as a result of enhanced microhardness, grain refinement, and beneficial compressive residual stress.

## 5. Conclusions

In the present work, we comprehensively explored the mechanical properties, microstructural evolution, and wear resistance of conventional metallic materials, laser additively manufactured parts, and laser cladding coatings treated with LSP. Below, we report our main findings:

(1) In metallic materials, LSP treatment imparts beneficial compressive residual stress to the material surface and improves the grain boundary structure, leading to grain refinement and enhanced grain boundary continuity, consequently augmenting the material’s mechanical properties.
(2) The application of LSP enhances the microhardness and wear resistance of the surface of cast and forged parts, additively manufactured components, and laser cladding coatings and reduces their COF and wear rate. Additionally, LSP treatment effectively eliminates surface cracks and defects, consequently enhancing the overall quality of the components.
(3) The wear resistance enhancement mechanism of metallic materials treated by using LSP effectively reduces the infiltration of abrasive grains and minimizes both abrasive wear and fatigue wear. Furthermore, LSP treatment effectively eliminates internal defects and stress within metals, thereby enhancing their overall structural stability and durability.

## Figures and Tables

**Table 1:** Advantages and drawbacks of different confining layer materials in industrial applications [7].

Confining Layer Material	Advantages	Drawbacks	Application Scenario
Running water	Cost effectiveness	Unsuitability for use at high temperatures	Large-scale, room-temperature LSP
	High availability		
	Safety	Difficult-to-control thickness	
	High flexibility		
Glass	Heat resistance (≥500 °C)	Inflexibility	Small-scale LSP processing in laboratory settings
	High shock impedance High optical transparency	Proneness to surface cracks and breaking	
Glycerol	Heat resistance (≥300 °C)	High viscosity and poor flow	Large-scale, high-temperature sample processing
	Insulation	Lack of safety	

**Table 2:** Average mass loss results of experimental specimens before and after both LSP treatment and friction test [10].

		Before LSP	After LSP
Number of samples		3	5
Average mass per sample (g)	Before test	27.1725	27.3241
	After test	27.1571	27.3232
Mass loss per sample (mg)		15.4 (RSD = 7.5%)	0.9 (RSD = 28.2%)
Decrease in average mass loss (%)		94	

**Table 3:** Microhardness of Ti-6Al-7Nb specimen before and after LSP treatment [45].

Parameter	Untreated Specimen	LSP 1	LSP 2	LSP 3
Surface microhardness (HV)	310	363.2	367.5	372.2
Work hardening depth (mm)	/	0.9	0.9	0.9

**Table 4:** Residual stress and microhardness of 15-5PH stainless steel specimens before and after LSP treatment [21].

Parameter	Before LSP Treatment	After LSP Treatment
Surface residual stress (MPa)	122	−340
Surface microhardness (HV)	373.5	422.3
Compress residual stress depth (mm)	/	0.74
Work hardening depth (mm)	/	1

**Table 5:** Residual stress and microhardness values of experimental specimens before and after LSP treatment [59].

Parameter	As-Machined Specimen	LC-Treated Specimen	LSP LC-Treated Specimen
Surface residual stress (MPa)	0	167	−453
Surface microhardness (HV)	250	432	550
Compress residual stress depth (mm)	/	/	1
Work hardening depth (mm)	/	/	0.8

## Data Availability

All data supporting the conclusions of this manuscript are included within the manuscript.
